# Outcomes of bicanalicular nasal stent inserted by sheath-guided dacryoendoscope in patients with lacrimal passage obstruction: a retrospective observational study

**DOI:** 10.1186/s12886-020-01678-5

**Published:** 2021-02-25

**Authors:** Tomoyuki Kamao, Xiaodong Zheng, Atsushi Shiraishi

**Affiliations:** Department of Ophthalmology, Ehime University Graduate School of Medicine, Shitsukawa, Toon, Ehime 791-0295 Japan

**Keywords:** Lacrimal passage obstruction, Lacrimal stent, Dacryoendoscope, Bicanalicular intubation, Glasgow benefit inventory

## Abstract

**Background:**

The dacryoendoscope is the only instrument that can observe the luminal side of the lacrimal passage with minimal invasiveness. It was developed to treat lacrimal passage obstructions by inserting a bicanalicular nasal stent with sheath-guided bicanalicular intubation (SG-BCI). The purpose of this study was to determine the outcomes of SG-BCI to treat lacrimal passage obstructions. In addition, to determine the effects of SG-BCI treatment on the quality of life.

**Methods:**

This was a retrospective observational study of 128 patients (mean age 70.9 ± 11.0 years, range 28–93 years) diagnosed with a unilateral lacrimal passage obstruction. There were 73 patients with a nasolacrimal duct obstruction, 37 with a lacrimal canaliculus obstruction, 7 with a lacrimal punctum obstruction, and 11 with common lacrimal canaliculus and nasolacrimal duct obstructions. They were all treated with SG-BCI. The postoperative subjective outcomes were assessed by the answers to the Glasgow Benefit Inventory (GBI) questionnaire and to an ocular specific questionnaire on 6 symptoms including tearing, ocular discharges, swelling, pain, irritation, and blurred vision. The objective assessments were the surgical success rates and the patency at 6 months after the bicanalicular nasal stent was removed. The patients were divided into those with a pre-saccal obstruction, Group 1, and with a post-saccal obstruction, Group 2. The subjective and objective outcomes were compared between the two groups.

**Results:**

One hundred twenty-four sides (96.9%) had a successful probing and intubation of the lacrimal passage obstruction by SG-BCI. Of the 124 sides, 110 sides (88.7%) retained the patency after the stent was removed for at least 6 months. The GBI total, general subscale, social support, and physical health scores were + 37.1 ± 29.0, + 41.5 ± 30.0, + 28.0 ± 39.4, and + 24.1 ± 37.7, respectively, postoperatively. All of the 6 ocular specific symptom scores improved significantly postoperatively. The postoperative score of tearing improved in Group 1 (*P* < 0.0001), while the postoperative scores of all symptoms improved significantly in Group 2.

**Conclusions:**

The relatively high surgical success rates and positive GBI scores, and improved ocular symptom scores indicate that SG-BCI is a good minimally invasive method to treat lacrimal passage obstructions.

## Background

Epiphora is a common complaint in patients with a lacrimal passage obstruction visiting ophthalmology clinics. The obstructions have been treated by external dacryocystorhinostomy (EX-DCR) as first described by Toti in 1904, and this has been the gold standard treatment for a primary acquired nasolacrimal duct obstruction (PANDO) with success rates ranging from 90 to 99% [1, 2]. Endonasal dacryocystorhinostomy (EN-DCR) was first described by Killian [3] and Caldwell [4]. Later, McDonogh and Meiring introduced a nasal endoscopic technique for DCR [5], and EN-DCR has increased in popularity over the past several decades. The recent development of nasal endoscopic techniques and instruments have led to higher success rates for EN-DCR to treat PANDOs [2, 6–8].

The technique of bicanalicular intubation (BCI) to treat PANDOs was introduced as an alternative to DCR [9–13]. Although modifications of the original technique have increased the success rates for BCI, the success rate of 22.2–79.5% is still lower than that of DCR [12–19]. One of the reasons for the lower success rates might be because inserting the lacrimal stent is a blind technique without visual guidance. In addition, there are large variations in the morphology of the lacrimal passage [20]. As best we know, there has been only one report comparing the success rates for BCI of the blind insertion to dacryoendoscope-assisted intubation. Thus, Fujii et al. reported that 87.8% of the nasolacrimal ducts that had dacryoendoscope-assisted intubation remained patent, while 71.4% of the nasolacrimal ducts that were intubated without a dacryoendoscope remained patent at 1 month after the stent was removed [21].

To overcome this problem, a dacryoendoscope was developed that allowed a direct visualization of the luminal side of the lacrimal passage during the dislodgement of the occlusion and the insertion of a lacrimal stent [5, 22–25]. The dacryoendoscope has been shown to be a useful instrument not only for observing the lacrimal passage but also for treating lacrimal passage obstructions [9, 25–29]. The treatment of lacrimal passage obstructions using a dacryoendoscope has increased the success rates. Sugimoto and Inoue developed a sheath-guided endoscopic probing and intubation technique for BCI (SG-BCI), and its use has led to success rates of 70 to 90% for treating PANDOs although it is still lower than DCR [27–29].

The assessments of the treatment outcomes have been based on the postoperative improvements of the signs and symptoms caused by the lacrimal passage obstructions. Thus, after the dacryoendoscope was introduced to treat lacrimal passage obstructions, an improvement in the postoperative signs have been reported but the postoperative symptoms have not been well evaluated [27–29].

The Glasgow Benefit Inventory (GBI) is a comprehensive, verified post-interventional questionnaire which evaluates the influences of an intervention on the quality of life (QOL) of individuals [30]. It has been used to assess the patients’ perception of the benefits after undergoing different DCR techniques [31–36]. As best we know, GBI has not been used in patients who had undergone SG-BCI.

Thus, the purpose of this study was to determine the outcomes of SG-BCI objectively and subjectively. To accomplish this, we determined whether the changes in the symptoms and QOL of the patients improved, and we also compared the ocular symptoms scores before the treatment to that after the stent was removed.

## Methods

### Study design

This was a retrospective observational cohort study designed to assess the success of SG-BCI on patients with lacrimal passage obstructions. In addition, the QOL was assessed by the GBI questionnaire, and the symptoms were assessed by the ocular symptom scores.

### Subjects

One hundred twenty-eight patients whose mean age was 70.9 ± 11.0 years with a range 28–93 years were studied. There were 37 (28.9%) men and 91 (71.1%) women. All were diagnosed with unilateral lacrimal passage obstruction at the Ehime University Hospital between December 2010 and May 2014 and were examined and treated by 2 ophthalmologists (TK, AS). The diagnosis of a lacrimal passage obstruction was based on the dye disappearance test, lacrimal irrigation, lacrimal cannulation, and dacryoendoscopic examinations. None of the patients had a history of acute dacryocystitis or functional nasolacrimal duct obstruction.

### Treatment protocols

**Fig. 1:**
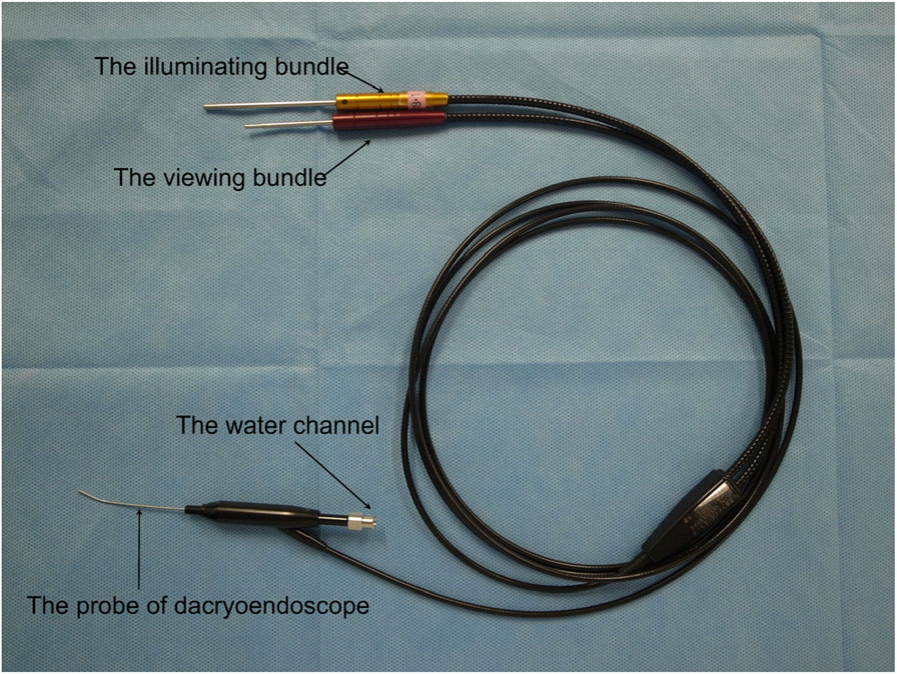
Photograph of a dacryoendoscope. The dacryoendoscope consists of a viewing and illuminating fiber optic bundle, and a water channel. The tip of the probe has an objective lens, light guide, and fluid nozzle. The outer diameter of the probe is 0.9 mm. The probe of the dacryoendoscope is bent at a 27-degree angle at 10 mm from its tip and has a 70-degree field of view

**Fig. 2:**
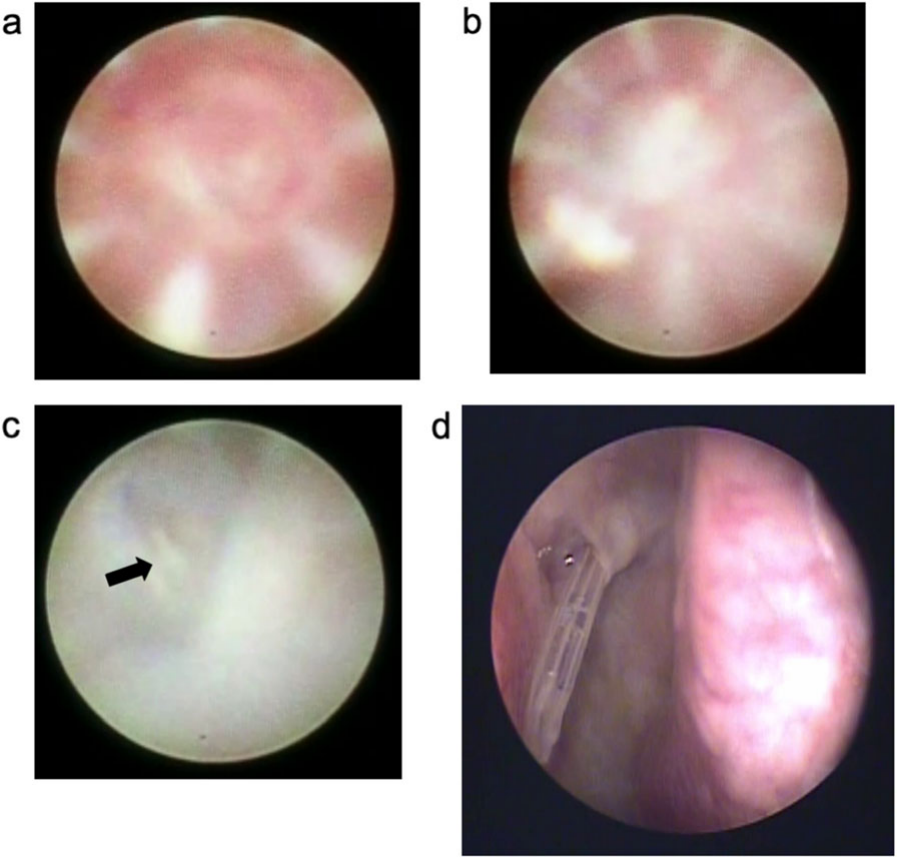
Intraoperative view with a dacryoendoscope and nasal endoscope. The white fibrous lesion in the center is the occluded site (**a**). The tip of an 18-gauge catheter is placed on the occluded site (**b**). The occlusion is dislodged by sheath-guided endoscopic probing with the tip of the 18-gauge catheter. The gauze inserted in the inferior meatus can be seen (black arrow) (**c**). The view through the nasal endoscope at the inferior meatus. The right side is the inferior turbinate and left side is the inferior meatus. An 18-gauge catheter is seen passing through the opening of the nasolacrimal duct (**d**)

**Fig. 3:**
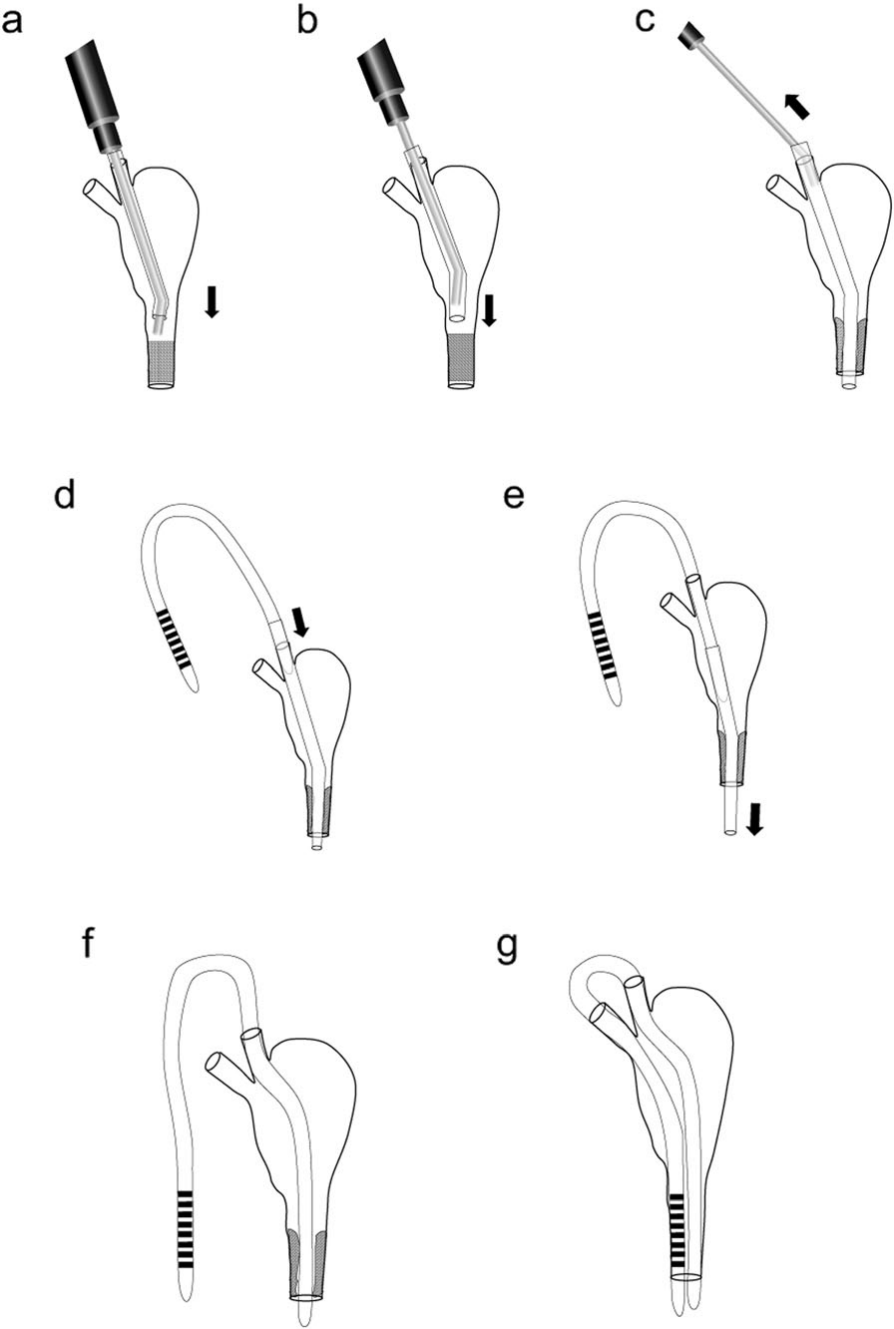
Sheath-guided endoscopic probing and intubation technique for bicanalicular intubation. The bicanalicular nasal stent is inserted by the sheath-guided bicanalicular intubation technique. The dacryoendoscope loaded with an 18-gauge catheter reaches the obstruction site, the obstruction is released with the tip of the dacryoendoscope (**a**) or with the tip of an 18-gauge catheter (**b**). After the dacryoendoscope with the 18-gauge catheter is reached the inferior meatus, the 18-gauge catheter is left in the inferior meatus and the lacrimal duct and then the dacryoendoscope is drawn out of the lacrimal duct (**c**). After a bicanalicular nasal stent is connected with the 18-gauge catheter on the lacrimal punctum side (**d**), the catheter is withdrawn from the lacrimal duct through the inferior meatus (**e**), to be able to draw the bicanalicular nasal stent into the recanalized lacrimal duct (**f**). The same procedure is performed on the other lacrimal punctum, and then the lacrimal passage obstruction is completely intubated (**g**). Figure was adapted from Kamao (2020) [37]

All patients were treated with the SG-BCI as described in briefly [37]. To explain in detail, the procedures were performed under topical anesthesia with 4% lidocaine, infratrochlear anesthesia with 2% lidocaine, and nasolacrimal anesthesia by 2% lidocaine and 0.1% noradrenalin. After dilation of the upper and lower lacrimal puncta by a punctal dilator, the dacryoendoscope (FT-201, Fibertech, Tokyo, Japan, Fig. 1) loaded with an 18-gauge catheter (Terumo, Tokyo, Japan) as a sheath, was inserted through the upper or lower lacrimal punctum. Saline was injected through the water channel to obtain a clearer view of the lumen. When the dacryoendoscope reached the pre- or post-saccal obstruction (Fig. 2a), the obstruction was released with the tip of the dacryoendoscope (direct endoscopic probing technique) (Fig. 3a) or with the tip of the 18-gauge catheter (sheath-guided endoscopic probing technique) (Figs. 2b, c, 3b) [29]. After releasing the occluded site, the dacryoendoscope with the 18-gauge catheter was reached the inferior meatus. The 18-gauge catheter was left in the inferior meatus and the lacrimal duct and then the dacryoendoscope was drawn out of the lacrimal duct (Figs. 2d, 3c).

After a bicanalicular nasal stent, whose outer diameter at the tip was 1.0 mm (PF catheter, NIDEK, Gamagori, Japan), was connected with the 18-gauge catheter on the lacrimal punctum side (Fig. 3d), the catheter was withdrawn using nasal endoscope from the lacrimal duct through the inferior meatus (Fig. 3e) to be able to draw the bicanalicular nasal stent into the recanalized lacrimal duct (Fig. 3f). The same procedure was performed on the other lacrimal punctum, and then the lacrimal passage obstruction was completely intubated (Fig. 3g). After the SG-BCI, all patients were prescribed topical 0.1% fluorometholone and 0.3% gatifloxacin 4 times/day. Irrigation with saline to clear the lacrimal duct was applied regularly until the stent was removed. The bicanalicular nasal stent was removed after 10–12 weeks.

### Postoperative outcome assessments

To evaluate the outcomes objectively, the rate of patency, i.e., retained patency after the stent was removed for at least 6 months, was compared for each obstructed site, the severity of the blockage, and the duration of the epiphora. The severity of the blockage was classified as complete or partial obstruction. A complete obstruction was defined as one with no patent irrigation with a regurgitation of clear or mucoid fluid. A partial obstruction was defined as that when there is some regurgitation of the fluid. The duration of epiphora was based on patients’ report, and it was classified into three stages according to an earlier report [38]. Stage 1 had a short-term epiphora that had developed within 1 years preoperatively. Stage 2 had an intermediate duration epiphora with onset from 1 to 3 years. And Stage 3 had long-term duration epiphora that had developed more than 3 years earlier. In addition, the preoperative tear meniscus height (TMH) was compared to the postoperative TMH. These measurements were made by swept-source anterior segment optical coherence tomography (SS-1000 CASIA, Tomey, Nagoya, Japan) with a built-in software program as described in detail [39].

To evaluate the subjective outcomes, the ocular symptom questionnaire was used as described in detail [37]. Briefly, six symptoms including the excessive tearing, ocular discharges, swelling, pain, irritation, and blurred vision were selected according to two earlier reports [40, 41], and assessed by their frequency and the severity with a numeric scale from 0 to 4, respectively. The final symptom score was the sum of frequency and severity scores and they ranged from 0 to 8. The assessments were made preoperatively and at 6 months after the stent was removed.

The GBI questionnaire was used to evaluate the changes in the QOL including changes in the psychological and social functions following the DCR (Additional file 1) [30]. The GBI questionnaire consisted of 18 items each ranging from − 100 (maximal negative benefit) through 0 (no changes) to + 100 (maximal positive benefit). It was scored by the total score and three subscores which consisted of a general subscale score (12 items), a social support score (3 items), and a physical health score (3 items). The patients answered the GBI questionnaire 6 months after the bicanalicular nasal stent was removed.

### Statistical analyses

Data were analyzed with the JMP software ver. 11.2 (SAS Institute, Cary, North Carolina, USA). Paired *t-*tests were used to compare TMH between the pre- and postoperative findings. Wilcoxon signed-rank tests were used to compare the preoperative ocular symptom scores to that at 6 months after the stent was removed. To evaluate the subjective outcomes more accurately, the subjects were divided into two groups by the site of obstruction. Patients with an obstruction of the lacrimal punctum, the lacrimal canaliculus, or common lacrimal canaliculus, i.e., pre-saccal obstruction, were placed in Group 1. Patients with an obstruction after the lacrimal sac, i.e., post-saccal obstruction, were placed in Group 2. The ocular symptom scores were evaluated by Mann-Whitney’s U tests, and the GBI scores were evaluated by Student *t*-tests between the Group 1 and the Group 2. A *P* value of < 0.05 was considered statistically significant.

## Results

### Assessments of objective outcomes

The demographics of the 128 patients are presented in Table 1. Of the 128 patients, 73 had a nasolacrimal duct obstruction (22 lacrimal sac, 33 proximal, 18 distal), 37 had a lacrimal canaliculus obstruction, 7 had a lacrimal punctum obstruction, and 11 had common lacrimal canaliculus and nasolacrimal duct obstructions. The mean duration of the stent placement was 88.7 ± 24.6 days, and the mean observation period after removing the bicanalicular nasal stent was 16.3 ± 12.0 months. Among the 128 patients, 124 (96.9%) had surgical success and 4 (3.1%) had a surgical failure, i.e., not able to probe correctly and not able to insert the bicanalicular nasal stent because the occluded site was too difficult to remove, or a false passage was made because of severe fibrosis at the obstruction site. The failed sides included 3 (8.1%) lacrimal canaliculus obstructions and 1 (3.0%) proximal nasolacrimal duct obstruction. The TMH was significantly decreased in the both obstructed site group except for the lacrimal punctum and common lacrimal canaliculus and nasolacrimal duct obstructions groups. There were no significant differences in the age, sex distribution, intubation period, observation period, TMH, and surgical success rate among the obstructed site groups.

**Table 1:** Demographics of 128 patients who were enrolled the study

Obstructed site	Number	Age	Sex (male/ female)	Intubation perriod (day)	Obseravation period (month)	TMH (μm)		*P* value	surgical success (%)	patency (%)
						Pre	Post			
All patients	128	70.9 ± 11.0	37/91	88.7 ± 24.6	16.3 ± 12.0	0.448 ± 0.288	0.253 ± 0.164	*p* < 0.0001	124 (96.9)	110 (88.7)
Lacrimal punctum	7	61.7 ± 20.8	2/5	101.6 ± 63.8	7.3 ± 1.2	0.672 ± 0.265	0.479 ± 0.157	*p* = 0.1527	7 (100.0)	7 (100.0)
Lacrimal canaliculus	37	71.6 ± 9.8	12/25	82.1 ± 13.6	17.5 ± 11.8	0.494 ± 0.272	0.268 ± 0.205	*p* = 0.0003	34 (91.9)	31 (91.2)
Common lacrimal canaliculus and nasolacrimal duct	11	70.3 ± 11.5	2/9	74.9 ± 14.8	23.2 ± 10.7	0.403 ± 0.211	0.208 ± 0.107	*p* = 0.0944	11 (100.0)	11 (100.0)
Lacrimal sac	22	74.8 ± 8.9	4/18	89.5 ± 12.6	20.2 ± 14.3	0.469 ± 0.415	0.236 ± 0.125	*p* = 0.0096	22 (100.0)	17 (77.3)
Proximal nasolacrimal duct	33	69.8 ± 10.8	8/25	93.1 ± 18.8	13.8 ± 11.6	0.380 ± 0.224	0.239 ± 0.112	*p* = 0.0013	32 (97.0)	29 (90.6)
Distal nasolacrimal duct	18	70.5 ± 9.5	9/9	96.9 ± 38.2	12.2 ± 9.0	0.467 ± 0.257	0.251 ± 0.124	*p* = 0.0036	18 (100)	15 (83.3)

*P* values were determined with a paired *t*-test. TMH tear meniscus height, “Pre” indicates preoperatively. “Post” indicates 6 months after the removal of the stent

In the 124 surgically successful sides, 14 sides (11.3%) had a recurrence and 110 sides (88.7%) retained patency. The rate of patency for each obstructed site was 100.0% (7/7) for lacrimal punctum obstruction, 91.2% (31/34) for lacrimal canaliculus obstruction, 100.0% (11/11) for common lacrimal canaliculus and nasolacrimal duct obstructions, 77.3% (17/22) for lacrimal sac obstruction, 90.6% (29/32) for proximal nasolacrimal duct obstruction, and 83.3% (15/18) for distal nasolacrimal duct obstruction. The rate of patency was not significantly different between the pre- (Group 1) and post-saccal obstruction groups (Group 2).

Next, the rate of patency was classified by the severity of the blockage (Table 2). The results showed that 95.6% (43/45) with complete obstruction and 90.0% (9/10) with partial obstruction remained patent in sides with pre-saccal obstructions. In sides with post-saccal obstructions, 83.1% (54/65) with complete obstructions and 100.0% (8/8) with partial obstruction remained patent. Then, the rate of patency was classified by the duration of epiphora. For sides with pre-saccal obstructions, 92.9% (26/28), 100.0% (10/10), and 91.7% (11/12) retained patency for stage 1, 2, and 3 epiphora respectively (Table 3). For post-saccal obstructions, 90.9% (30/33), 82.4% (14/17) and 77.3% (17/22) retained patency at stage 1, 2, and 3, respectively.

**Table 2:** The rate of patency was classified into severity of blockage

	Severity	Number	Patency (%)
Pre-saccal	Complete obstruction	45	43 (95.6)
	Partial obstruction	10	9 (90.0)
Post-saccal	Complete obstruction	65	54 (83.1)
	Partial obstruction	8	8 (100.0)

**Table 3:** The rate of patency was classified into duration of epiphora

	Duration	Number	Patency (%)
Pre-saccal	Stage 1	28	26 (92.9)
	Stage 2	10	10 (100.0)
	Stage 3	12	11 (91.7)
Post-saccal	Stage 1	33	30 (90.9)
	Stage 2	17	14 (82.4)
	Stage 3	22	17 (77.3)

Stage 1 has short-term epiphora that had developed within 1 years preoperatively. Stage 2 has intermediate epiphora with onset from 1 to 3 years. Stage 3 has long-term epiphora that had developed more than 3 years

### Assessments of subjective outcomes

Of the 128 cases, subjective assessments were completed in 91 patients consisting of 23 men and 68 women. The demographics of these 91 patients are shown in Table 4. The mean age of the 91 patients was 69.7 ± 10.4 years with a range of 29 to 92 years. The obstructed site was the nasolacrimal duct on 56 sides (61.5%) (17 lacrimal sac, 23 proximal, 16 distal nasolacrimal duct obstructions), lacrimal canaliculus on 27 sides (29.7%), lacrimal punctum on 5 sides (5.5%), and common lacrimal canaliculus and nasolacrimal duct on 3 sides (3.3%). The mean duration of the stent placement was 97.8 ± 58.2 days, and the mean observation period after removal of the bicanalicular nasal stent was 15.7 ± 12.4 months. None of the 91 patients had a recurrence at 6 months after the stent was removed.

**Table 4:** Demographics of 91 patients who were completed subjective assessments

Obstructed site	Number	Age	Sex (male/ female)	Intubation perriod (day)	Obseravation period (month)	TMH (μm)		*P* value
						Pre	Post	
All patients	91	69.7 ± 10.4	23/68	97.8 ± 58.2	15.7 ± 12.4	0.429 ± 0.274	0.235 ± 0.151	*p* < 0.0001
Lacrimal punctum	5	54.0 ± 19.6	0/5	68.2 ± 19.9	6.0 ± 0.0	0.406 ± 0.154	0.311 ± 0.168	*p* = 0.3258
Lacrimal canaliculus	27	69.3 ± 9.8	8/19	91.0 ± 45.8	15.6 ± 10.3	0.497 ± 0.272	0.265 ± 0.206	*p* < 0.0001
Common lacrimal canaliculus and nasolacrimal duct	3	70.0 ± 10.0	0/3	199.7 ± 196.9	14.3 ± 8.5	0.403 ± 0.211	0.175 ± 0.092	*p* = 0.0944
Lacrimal sac	17	71.2 ± 7.9	4/13	86.9 ± 28.3	20.0 ± 16.6	0.468 ± 0.401	0.210 ± 0.122	*p* = 0.0028
Proximal nasolacrimal duct	23	72.1 ± 9.2	5/18	88.0 ± 10.7	16.6 ± 13.9	0.351 ± 0.226	0.212 ± 0.109	*p* = 0.0014
Distal nasolacrimal duct	16	70.1 ± 9.1	6/10	102.2 ± 37.8	11.0 ± 7.3	0.393 ± 0.202	0.229 ± 0.117	*p* = 0.0055
Group 1	35	67.2 ± 12.4	8/27	106.5 ± 88.0	14.8 ± 10.0	0.476 ± 0.252	0.264 ± 0.193	*p* < 0.0001
Group 2	56	71.3 ± 8.7	15/41	92.7 ± 30.5	16.2 ± 13.7	0.399 ± 0.285	0.216 ± 0.114	*p* < 0.0001

*P* values were determined with a paired *t*-test. TMH tear meniscus height, “Pre” indicates preoperatively. “Post” indicates 6 months after the removal of the stent. Group 1 includes patients with an obstruction of the lacrimal punctum, the lacrimal canaliculus and both common lacrimal canaliculus and nasolacrimal duct. Group 2 includes an obstruction of the lacrimal sac, the proximal nasolacrimal duct and distal nasolacrimal duct

**Fig. 4:**
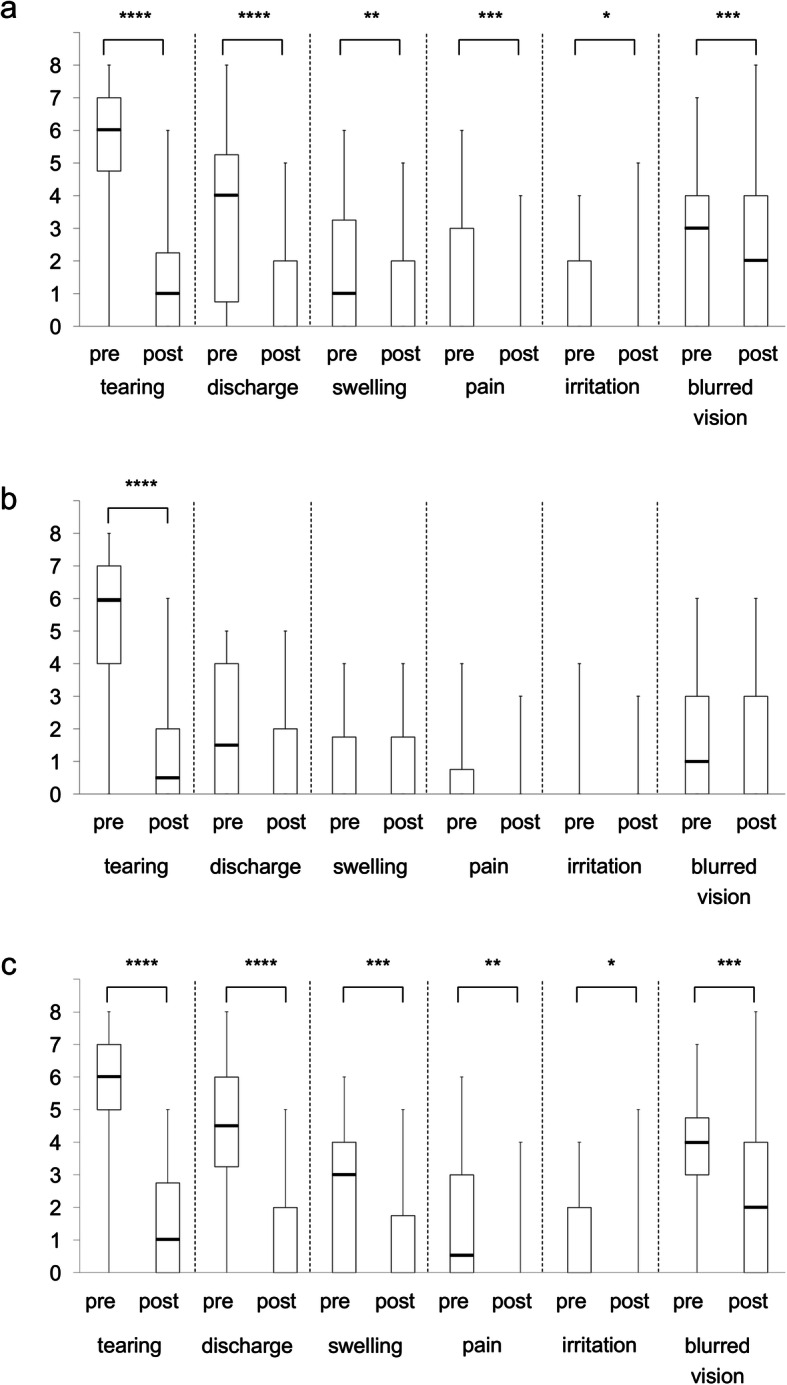
Ocular specific symptom scores. The horizontal line in the middle of each box indicates the median, while the top and bottom borders of the box mark the 75th and 25th percentiles, respectively. The whiskers above and below the box mark the minimum and maximum. “Pre” indicates preoperatively; and “post” indicates 6 months after the removal of the stent. Group 1, the pre-saccal obstruction group; Group 2, the post-saccal obstruction group. **a** Six ocular specific symptom scores for the preoperatively and 6 months after the removal of the stent. **b** The scores for Group 1. **c.** The scores for Group 2. Wilcoxon signed-rank tests were used to compare the pre- and the post-saccal groups. *P < 0.05; ** *P* < 0.01; *** *P* < 0.001; **** *P* < 0.0001

An analyses of the outcomes showed that all 6 ocular symptom scores improved significantly compared to the preoperative scores at 6 months after the stent was removed; tearing and discharge (*P* < 0.0001), swelling (*P* = 0.0024), pain (*P* = 0.0002), irritation (*P* = 0.0456), and blurred vision (*P* = 0.0003; Fig. 4a).

At 6 months after the stent was removed, the mean GBI total score was + 37.1 ± 29.0, the general subscale score was + 41.5 ± 30.0, the social support score was + 28.0 ± 39.4, and the physical health score was + 24.1 ± 37.7 (Table 5). The GBI scores were positive for the total and three subset scores at 6 months after the stent was removed. These findings indicated that the patients had a marked benefit of the SG-BCI technique.

**Table 5:** GBI scores at 6 months after the stent was removed

	All patients (mean ± SD)	Group 1 (mean ± SD)	Group 2 (mean ± SD)	*P* Value
Total score	+ 37.1 ± 29.0	+ 39.4 ± 32.8	+ 37.4 ± 29.7	0.5139
General subscale score	+ 41.5 ± 30.0	+ 45.4 ± 32.6	+ 43.1 ± 29.2	0.4339
Social support score	+ 28.0 ± 39.4	+ 26.2 ± 40.3	+ 28.5 ± 41.5	0.9776
Physical health score	+ 24.1 ± 37.7	+ 29.4 ± 42.5	+ 21.1 ± 37.3	0.3381

*P* values were determined with a Standard *t*-test. SD indicates standard deviation

The postoperative score for tearing improved significantly (*P* < 0.0001, Fig. 4b) in Group 1 while all 6 of the postoperative ocular symptom scores improved significantly in Group 2, e.g., tearing and discharge (*P* < 0.0001), swelling (*P* = 0.0005), pain (*P* = 0.0011), irritation (*P* = 0.0285), and blurred vision (*P* = 0.0002; Fig. 4c).

**Fig. 5:**
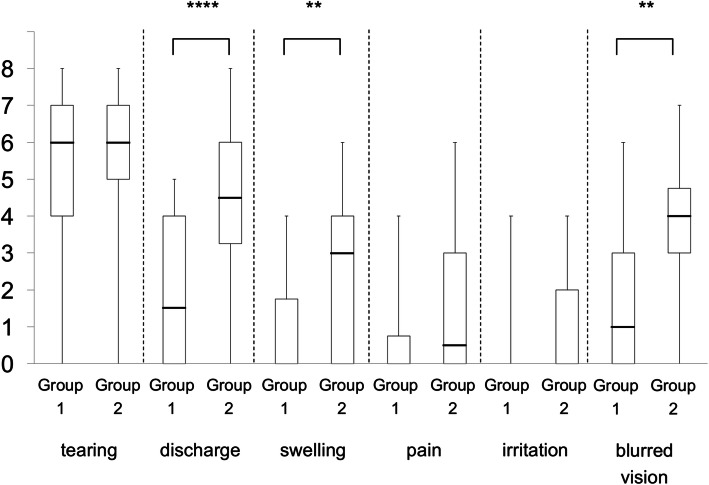
Ocular specific symptom scores of pre-saccal and post-saccal obstruction for the preoperatively assessments. The horizontal line in the middle of each box indicates the median, while the top and bottom borders of the box mark the 75th and 25th percentiles, respectively. The whiskers above and below the box mark the minimum and maximum. Group 1, pre-saccal obstruction group; Group 2, post-saccal obstruction group. Mann-Whitney’s U tests were used to compare between Group 1 and 2. ** *P* < 0.01; **** *P* < 0.0001

The preoperative symptom scores of tearing, pain, and irritation were not significantly different between the two groups, however the scores in Group 2 were significantly higher than that in Group 1 for discharge (*P* < 0.0001), swelling (*P* = 0.0068), and blurred vision (*P =* 0.0010; Fig. 5). The postoperative symptom scores of all 6 symptoms were not significantly different between the two groups (data not shown).

The mean GBI total score was + 39.4 ± 32.8, the general subscale score was + 45.4 ± 32.6, the social support score was + 26.2 ± 40.3, and the physical health score was + 29.4 ± 42.5 in Group 1. The mean GBI total score was + 37.4 ± 29.7, the general subscale score was + 43.1 ± 29.2, the social support score was + 28.5 ± 41.5, and the physical health score was + 21.1 ± 37.3 in Group 2 (Table 5). No significant differences were found in the total and three subsets GBI score between Groups 1 and 2.

## Discussion

Dacryoendoscopy was first used for bicanalicular nasal stent insertion in cases with lacrimal passage obstructions, and relatively favorable outcomes of the dacryoendoscope-assisted intubation have been reported. Sugimoto et al. reported that 90% of patients with lacrimal canalicular obstruction and 64% of patients with nasolacrimal duct obstructions that had dacryoendoscope-assisted intubation remained patent for 8.4 years after the surgery [27]. In our study, 100% of the patients with lacrimal punctum obstruction, 91.2% with lacrimal canaliculus obstruction, 100% with common lacrimal canaliculus and nasolacrimal duct obstructions, 77.3% with lacrimal sac obstruction, 90.6% with proximal nasolacrimal duct obstruction, and 83.3% with distal nasolacrimal duct obstruction, that had undergone the SG-BCI procedure remained patent for an average of 16.3 ± 12.0 months after the stent was removed. No significant difference was seen for each obstructed site. However, Sugimoto et al. reported that the outcome of lacrimal canalicular obstruction was better than that of nasolacrimal obstruction. These differences may be due to the difference in the duration of the follow-up period and the severity of the blockage. Thus, the SG-BCI technique has a relatively high success rate and is a minimally invasive surgery which explains why this technique has become a commonly used treatment for lacrimal passage obstructions in Japan.

To evaluate the treatment outcomes more intensively, the subjects were divided by the severity of the blockage and the duration of the epiphora. The outcomes were not significantly different between the complete and partial obstructions in the pre-saccal group. On the other hand, the patency rate in sides with a complete obstruction was significantly lower than that with partial obstruction of the post-saccal group. The same tendency was found in the outcomes for the duration of the epiphora, i.e., the outcomes were not significantly different for the three stages for pre-saccal obstructions. However, sides with longer durations epiphora were associated with a lower rate of patency for post-saccal obstructions.

We conclude that SG-BCI was more effective for pre-saccal obstructions than for post-saccal obstructions. These results are consistent with previous reports [11, 15, 18, 27]. Mimura et al. suggested two reasons for these outcomes. First, pre-saccal obstructions have a lower chance for severe or chronic inflammation such as dacryocystitis or rhinitis, than post-saccal obstructions. Second, the anatomical and histological differences between the pre-saccal and post-saccal parts affect the rate of patency of SG-BCI. The length of mucosal damage with obstruction or stenosis in the pre-saccal part can be shorter than that in the post-saccal part anatomically. Moreover, the pre-saccal part which is lined by nonkeratinizing stratified squamous epithelium re-epithelializes easier than the post-saccal part which is lined by stratified columnar epithelium. These differences may lead to a higher rate of patency for pre-saccal obstructions than for post-saccal obstruction.

The outcomes of SG-BCI for post-saccal obstructions depended on the severity and duration of the obstruction. We suggest that the reason for these effects is because severe fibrosis of the mucosa caused by the inflammation can make the re-epithelialization more difficult even when it is reconstructed properly. Linberg et al. suggested that a long duration of PANDO causes severe and irreversible anatomical changes of the entire duct which then causes difficulties in reconstruction and remodeling [38]. Thus, we conclude that the early stages of post-saccal obstruction with mild mucosal damage can recover with reconstructive surgery such as SG-BCI, and the late stage of severe mucosal damage should be treated by bypass surgery such as by DCR.

The QOL following the BCI was assessed by the GBI questionnaire because GBI has been used to investigate the patients’ perception of the therapeutic effects of various DCR techniques [31–36]. The mean value for the total GBI score was + 37.1 in our study, while the score after DCR was found to range from + 15.04 to + 52 previously [2, 31–36, 41]. Although details of the patients’ demographics and surgical procedures varied among the different studies, the total GBI score in our study was comparable to those reported. This suggests that the results of the SG-BCI procedure led to comparable DCR improvements of the QOL.

However, the GBI was not specifically developed to evaluate the effects of treatments of ocular symptoms, and it has been demonstrated that the GBI was not able to determine slight changes in the QOL [31, 32, 41]. Furthermore, the GBI assessments may not be able to compare the changes in the symptoms between the preoperative and postoperative scores because the GBI assessments were done only postoperatively. Therefore, the ocular symptoms questionnaire was also applied to compare the subjective outcomes pre- and postoperatively. Our results revealed that all of the 6 symptoms were improved after the SG-BCI procedure. This suggested that the improvement of ocular symptoms most likely led to the improvement of the QOL.

Although all 6 items were improved after the SG-BCI procedure, the severity of the symptoms probably varied depending on the obstruction site because dacryocystitis and conjunctivitis often develops in patients with nasolacrimal duct obstruction but not in the patients with lacrimal canalicular obstructions. Thus, the subjects were divided into pre-saccal and post-saccal obstruction groups, and the subjective outcomes were evaluated more precisely. The preoperative scores for discharges, swelling, and blurred vision were significantly higher in the post-saccal obstruction group than in the pre-saccal group (Fig. 5). These factors represent the complications of dacryocystitis and conjunctivitis in patients with nasolacrimal duct obstructions. Only the postoperative scores of tearing improved in the pre-saccal obstruction group (Fig. 4b) indicating the low rates of complications associated with dacryocystitis and conjunctivitis caused by lacrimal canalicular obstructions. In the post-saccal obstruction group, all of the 6 symptoms were improved postoperatively including tearing (Fig. 4c). These findings confirmed that epiphora is a major symptom in patients with lacrimal passage obstruction, and that the improvement of tearing is one of the main factors that contributed to the improvement of the QOL.

This study has some limitations. First, the retrospective and single facility design limited our ability to draw definitive conclusions in assessing the QOL of the patients that had undergone SG-BCI. Second, the postoperative observation period was relatively short to assess the objective surgical success rates and subjective assessments because of the study design to assess the outcomes by a questionnaire 6 months after the removal of the tube. A multicenter study is needed to evaluate the outcomes in a prospective long-term study before these findings can be fully validated. Third, the scores of the questionnaire and GBI did not include surgical failures or recurrences because of the study design to assess the questionnaire and GBI 6 months after the removal of the tube. Further studies are needed to determine the scores including surgical failures and recurrences. Moreover, a recall bias may have been present in this questionnaire because this questionnaire is not of standard format. Other assessments, for example Lac-Q [42] and Ocular Surface Diseased Index [43] were not used in this study. In addition, the subjects in this study were primarily Japanese which limits our ability to draw conclusions on the usefulness of SG-BCI on other races or ethnicities. Because of the limited shape of the dacryoendoscope, it may not be suitable for facial or lacrimal duct configurations of all races and ethnicities.

## Conclusions

In conclusion, our results indicated a relatively high surgical success rate, positive GBI scores, and improved ocular symptoms scores after the SG-BCI for lacrimal passage obstructions. The results indicate that the SG-BCI technique is an effective option for the treatment of lacrimal passage obstructions.

## Supplementary information

**Additional file 1.** The GBI questionnaire. Total Score: Sum all the responses (Qu. 1–18). Divide by 18 (to obtain an average response score). Subtract 3 from the average response score. Multiply by 50. General Subscale Score: Sum 12 of the responses (Qu. 1,2,3,4,5,6,9,10,14,16,17 and 18). Divide by 12 (to obtain an average response score). Subtract 3 from the average response score. Multiply by 50. Social Support Score: Sum 3 of the responses (Qu. 7,11,15). Divide by 3(to obtain an average response score). Subtract 3 from the average response score. Multiply by 50. Physical Health Score: Sum 3 of the responses (Qu. 8,12,13). Divide by 3(to obtain an average response score). Subtract 3 from the average response score. Multiply by 50.

## Data Availability

The datasets obtained and/or analyzed during the current study are available from the corresponding author on reasonable request.

## Abbreviations

SG-BCI: Sheath-guided bicanalicular intubation
GBI: Glasgow benefit inventory
EX-DCR: External dacryocystorhinostomy
PANDO: Primary acquired nasolacrimal duct obstruction
EN-DCR: Endonasal dacryocystorhinostomy
QOL: Quality of life
TMH: Tear meniscus height

## References

[1] Toti A (1904). Nuovo metodo conservatore di cura radicale delle suppurazioni croniche del sacco lacrimale (dacriocistorinostomia). Clin Mod.

[2] Hii BW, McNab AA, Friebel JD (2012). A comparison of external and endonasal dacryocystorhinostomy in regard to patient satisfaction and cost. Orbit.

[3] Killian J. Diskussion zu seiferts vortrag. Verhandlungen des Vereins Süddeutscher Laryngologen. 1899;6.

[4] Caldwell GW (1893). Two new operations for obstructions of the nasal duct with preservation of canaliculi and an incidental description of a new lacrimal probe. New York MJ.

[5] McDonogh M, Meiring JH (1989). Endoscopic transnasal dacryocystorhinostomy. J Laryngol Otol.

[6] Karim R, Ghabrial R, Lynch T, Tang B (2011). A comparison of external and endoscopic endonasal dacryocystorhinostomy for acquired nasolacrimal duct obstruction. Clin Ophthalmol.

[7] Lee DW, Chai CH, Loon SC (2010). Primary external dacryocystorhinostomy versus primary endonasal dacryocystorhinostomy: a review. Clin Exp Ophthalmol.

[8] Marcet MM, Kuk AK, Phelps PO (2014). Evidence-based review of surgical practices in endoscopic endonasal dacryocystorhinostomy for primary acquired nasolacrimal duct obstruction and other new indications. Curr Opin Ophthalmol.

[9] Kabata Y, Goto S, Takahashi G, Tsuneoka H (2011). Vision-related quality of life in patients undergoing silicone tube intubation for lacrimal passage obstructions. Am J Ophthalmol.

[10] Kurihashi K (1993). Bicanalicular silicone intubation using three-piece silicone tubing: direct silicone intubation. Ophthalmologica.

[11] Mimura M, Ueki M, Oku H, Sato B, Ikeda T (2015). Indications for and effects of Nunchaku-style silicone tube intubation for primary acquired lacrimal drainage obstruction. Jpn J Ophthalmol.

[12] Psilas K, Eftaxias V, Kastanioudakis J, Kalogeropoulos C (1993). Silicone intubation as an alternative to dacryocystorhinostomy for nasolacrimal drainage obstruction in adults. Eur J Ophthalmol.

[13] Soll DB (1978). Silicone intubation: an alternative to dacryocystorhinostomy. Ophthalmology.

[14] Anderson RL, Edwards JJ (1979). Indications, complications and results with silicone stents. Ophthalmology.

[15] Pashby RC, Rathbun JE (1979). Silicone tube intubation of the lacrimal drainage system. Arch Ophthalmol.

[16] Angrist RC, Dortzbach RK (1985). Silicone intubation for partial and total nasolacrimal duct obstruction in adults. Ophthalmic Plast Reconstr Surg.

[17] Liu D, Bosley TM (2003). Silicone nasolacrimal intubation with mitomycin-C: a prospective, randomized, double-masked study. Ophthalmology.

[18] Connell PP, Fulcher TP, Chacko E, Connor MJO, Moriarty P (2006). Long term follow up of nasolacrimal intubation in adults. Br J Ophthalmol.

[19] Demirci H, Elner VM (2008). Double silicone tube intubation for the management of partial lacrimal system obstruction. Ophthalmology.

[20] Narioka J, Matsuda S, Ohashi Y (2007). Correlation between anthropometric facial features and characteristics of nasolacrimal drainage system in connection to false passage. Clin Exp Ophthalmol.

[21] Fujii K, Inoue Y, Sugimoto M (2004). Outcome of endoscope-assisted silicone intubation for nasolacrimal duct obstruction. Rinsho Ganka (Jpn J Clin Ophthalmol).

[22] Ashenhurst ME, Hurwitz JJ (1991). Lacrimal canaliculoscopy: development of the instrument. Can J Ophthalmol.

[23] Cohen SW, Prescott R, Sherman M, Banko W, Castillejos ME (1979). Dacryoscopy. Ophthalmic Surg.

[24] Fein W, Daykhovsky L, Papaioannou T, Beeder C, Grundfest WS (1992). Endoscopy of the lacrimal outflow system. Arch Ophthalmol.

[25] Sasaki T, Nagata Y, Sugiyama K (2005). Nasolacrimal duct obstruction classified by dacryoendoscopy and treated with inferior meatal dacryorhinotomy: part II. Inferior meatal dacryorhinotomy. Am J Ophthalmol.

[26] Sasaki T, Sounou T, Sugiyama K (2009). Dacryoendoscopic surgery and tube insertion in patients with common canalicular obstruction and ductal stenosis as a frequent complication. Jpn J Ophthalmol.

[27] Sugimoto M, Inoue Y (2010). Long-term outcome of dacryoendoscope-assisted intubation for nasolacrimal duct obstruction. Atarashii Ganka (Journal of the Eye).

[28] Inoue Y (2008). New method of lacrimal passage intubation using teflon sheath as guide. Atarashii Ganka (Journal of the Eye).

[29] Sugimoto M (2007). New sheath-assisted dacryoendoscopic surgery. Atarashii Ganka (Journal of the Eye).

[30] Robinson K, Gatehouse S, Browning GG (1996). Measuring patient benefit from otorhinolaryngological surgery and therapy. Ann Otol Rhinol Laryngol.

[31] Bakri SJ, Carney AS, Robinson K, Jones NS, Downes RN (1999). Quality of life outcomes following dacryocystorhinostomy: external and endonasal laser techniques compared. Orbit.

[32] Ho A, Sachidananda R, Carrie S, Neoh C (2006). Quality of life assessment after non-laser endonasal dacryocystorhinostomy. Clin Otolaryngol.

[33] Jutley G, Karim R, Joharatnam N (2013). Patient satisfaction following endoscopic endonasal dacryocystorhinostomy: a quality of life study. Eye (Lond).

[34] Oh JR, Chang JH, Yoon JS, Jang SY (2015). Change in quality of life of patients undergoing silicone stent intubation for nasolacrimal duct stenosis combined with dry eye syndrome. Br J Ophthalmol.

[35] Spielmann PM, Hathorn I, Ahsan F, Cain AJ, White PS (2009). The impact of endonasal dacryocystorhinostomy (DCR), on patient health status as assessed by the Glasgow benefit inventory. Rhinology.

[36] Yeniad B, Uludag G, Kozer-Bilgin L (2012). Assessment of patient satisfaction following external versus transcanalicular dacryocystorhinostomy with a diode laser and evaluation if change in quality of life after simultaneous bilateral surgery in patients with bilateral nasolacrimal duct obstruction. Curr Eye Res.

[37] Kamao T, Takahashi N, Zheng X, Shiraishi A. Changes of visual symptoms and functions in patients with and without dry eye after lacrimal passage obstruction treatment. Curr Eye Res. 2020;3:1–8. 10.1080/02713683.2020.1760305 32321316

[38] Linberg JV, McCormick SA (1986). Primary acquired nasolacrimal duct obstruction. A clinicopathologic report and biopsy technique. Ophthalmology.

[39] Zheng X, Kamao T, Yamaguchi M (2014). New method for evaluation of early phase tear clearance by anterior segment optical coherence tomography. Acta Ophthalmol.

[40] Yokoi N, Komuro A, Nishii M (2005). Clinical impact of conjunctivochalasis on the ocular surface. Cornea.

[41] Smirnov G, Tuomilehto H, Kokki H (2010). Symptom score questionnaire for nasolacrimal duct obstruction in adults--a novel tool to assess the outcome after endoscopic dacryocystorhinostomy. Rhinology.

[42] Mistry N, Rockley TJ, Reynolds T, Hopkins C (2011). Development and validation of a symptom questionnaire for recording outcomes in adult lacrimal surgery. Rhinology.

[43] Schiffman RM, Christianson MD, Jacobsen G, Hirsch JD, Reis BL (2000). Reliability and validity of the ocular surface disease index. Arch Ophthalmol.

