# Supplementary material for: Outcomes of bicanalicular nasal stent inserted by sheath-guided dacryoendoscope in patients with lacrimal passage obstruction: a retrospective observational study
Source: BMC Ophthalmol. 2021 Feb 25;21:103. doi: 10.1186/s12886-020-01678-5 (PMC7905860; doi:10.1186/s12886-020-01678-5)
Supplement: Supplementary file 1 — Additional file 1. The GBI questionnaire. Total Score: Sum all the responses (Qu. 1–18). Divide by 18 (to obtain an average response score). Subtract 3 from the average response score. Multiply by 50. General Subscale Score: Sum 12 of the responses (Qu. 1,2,3,4,5,6,9,10,14,16,17 and 18). Divide by 12 (to obtain an average response score). Subtract 3 from the average response score. Multiply by 50. Social Support Score: Sum 3 of the responses (Qu. 7,11,15). Divide by 3(to obtain an average response score). Subtract 3 from the average response score. Multiply by 50. Physical Health Score: Sum 3 of the responses (Qu. 8,12,13). Divide by 3(to obtain an average response score). Subtract 3 from the average response score. Multiply by 50. [file 12886_2020_1678_MOESM1_ESM.pdf]

1. Has the result of the operation/intervention affected the things you do?

Much worse	A little or somewhat worse	No change	A little or somewhat better	Much better
1	2	3	4	5

2. Have the results of the operation/intervention made your overall life better or worse?

Much better	A little or somewhat better	No change	A little or somewhat worse	Much worse
5	4	3	2	1

3. Since your operation/intervention, have you felt more or less optimistic about the future?

Much more optimistic	More optimistic	No change	Less optimistic	Much less optimistic
5	4	3	2	1

4. Since your operation/intervention, do you feel more or less embarrassed when with a group of people?

Much more embarrassed	More embarrassed	No change	Less embarrassed	Much less embarrassed
1	2	3	4	5

5. Since your operation/intervention, do you have more or less self-confidence?

Much more self-confidence	More self-confidence	No change	Less self-confidence	Much less self-confidence
5	4	3	2	1

6. Since your operation/intervention, have you found it easier or harder to deal with company?

Much easier	Easier	No change	Harder	Much harder
5	4	3	2	1

7. Since your operation/intervention, do you feel that you have more or less support from your friends?

Much more support	More support	No change	Less support	Much less support
5	4	3	2	1

8. Have you been to your family doctor, for any reason, more or less often, since your operation/intervention?

Much more often	More often	No change	Less often	Much less often
1	2	3	4	5

9. Since your operation/intervention, do you feel more or less confident about job opportunities?

Much more confident	More confident	No change	Less confident	Much less confident
5	4	3	2	1

10. Since your operation/intervention, do you feel more or less self-conscious?

Much more self-conscious	More self-conscious	No change	Less self-conscious	Much less self-conscious
1	2	3	4	5

11. Since your operation/intervention, are there more or fewer people who really care about you?

Many more people	More people	No change	Fewer people	Many fewer people
5	4	3	2	1

12. Since you had the operation/intervention, do you catch colds or infections more or less often?

Much more often	More often	No change	Less often	Much less often
1	2	3	4	5

13. Have you had to take more or less medicine for any reason, since your operation/intervention?

Much more medicine	More medicine	No change	Less medicine	Much less medicine
1	2	3	4	5

14. Since your operation/intervention, do you feel better or worse about yourself?

Much better	Better	No change	Worse	Much worse
5	4	3	2	1

15. Since your operation/intervention, do you feel that you have had more or less support from your family?

Much more support	More support	No change	Less support	Much less support
5	4	3	2	1

16. Since your operation/intervention, are you more or less inconvenienced by your health problem?

Much more inconvenienced	More inconvenienced	No change	Less inconvenienced	Much less inconvenienced
1	2	3	4	5

17. Since your operation/intervention, have you been able to participate in more or fewer social activities?

Many more activities	More activities	No change	Fewer activities	Many fewer activities
5	4	3	2	1

18. Since your operation/intervention, have you been more or less inclined to withdraw from social situations?

Much more inclined	More inclined	No change	Less inclined	Much less inclined
1	2	3	4	5
